# Increased enhancement of the liver adjacent to the gallbladder seen with contrast ultrasound: comparison between acute cholecystitis and non-cholecystitis

**DOI:** 10.1186/s12880-016-0115-2

**Published:** 2016-03-10

**Authors:** Ryousuke Kawai, Jiro Hata, Noriaki Manabe, Hiroshi Imamura, Ai Iida, Nobuko Koyama, Hiroaki Kusunoki

**Affiliations:** Department of Clinical Pathology and Laboratory Medicine, Kawasaki Medical School, 577 Matsushima, Kurashiki, Okayama 701-0192 Japan; Department of Hepatology and Pancreatology, Kawasaki Medical School, 577 Matsushima, Kurashiki, Okayama 701-0192 Japan; Department of General Medicine, Kawasaki Medical School, 577 Matsushima, Kurashiki, Okayama 701-0192 Japan

**Keywords:** Acute cholecystitis, Contrast-enhanced ultrasound, Time-intensity curve analysis

## Abstract

**Background:**

This study was performed to evaluate the ability of contrast-enhanced ultrasonography (CEUS) with time-intensity curve analysis to demonstrate an increased enhancement of the liver parenchyma adjacent to the inflamed gallbladder, as seen on contrast-enhanced computed tomography.

**Methods:**

The Ethics Committee of our institution approved the study protocol (Kawasaki Medical School, registration number 1277). From April to November 2013, 11 consecutive patients with acute cholecystitis and 16 patients without cholecystitis consented to CEUS (Sonazoid™) and were enrolled in this study. The gallbladder and liver were scanned by one gastroenterologist using harmonic imaging with a low mechanical index. The raw imaging data were stored. Another physician, blinded to all clinical information, constructed the time-intensity curve. The major axis of the region of interest (ROI) was set in segment 5 (pericholecystic area), and the control ROI in segment 8 at the same depth. The intensity ratio (IR) was defined as the peak intensity of segment 5 divided by the simultaneous value of segment 8. The characteristics of the patient with and without acute cholecystitis were compared. The correlation between the IR and the presence of acute cholecystitis was analyzed using binomial logistic regression analysis. A receiver operating characteristic (ROC) curve analysis was performed as well.

**Results:**

The IR was significantly higher in the group with than without acute cholecystitis (*p* = 0.006). The IR correlated significantly with the presence of acute gallbladder inflammation (*p* = 0.043). The area under the ROC curve was estimated as 0.852 (95 % confidence interval, 0.709–0.995). A cut-off value of 2.72 had a sensitivity of 81.8 % and a specificity of 81.3 %.

**Conclusions:**

The IR obtained by CEUS with time-intensity curve analysis generally demonstrated increased enhancement of the liver parenchyma adjacent to the inflamed gallbladder.

## Background

Transabdominal ultrasonography (US) is regarded as the first-line noninvasive bedside examination for the diagnosis of acute abdominal diseases [1], including acute cholecystitis [2, 3], because it is safe, widely available, and inexpensive. Only patients with negative or inconclusive US findings should undergo computed tomography (CT), according to the general diagnostic strategy for acute abdominal pain, which aims for the highest sensitivity for urgent conditions and for the lowest radiation exposure [1]. Magnetic resonance imaging and cholescintigraphy are also useful for the diagnosis of acute cholecystitis [2, 3], although they are less available.

Imaging findings of gallbladder inflammation are needed for the diagnosis of acute cholecystitis according to the Tokyo Guideline 2013 (TG13) criteria [4, 5]. The reported sensitivity and specificity of gray-scale US are 88 % and 80 %, respectively [6]. However, typical imaging findings are not necessarily demonstrated in all cases. In our opinion, the diagnostic power of gray-scale US should be based on the patient’s specific complaints and should especially include the presence of a sonographic Murphy sign. For example, in a surgical series of patients with gangrenous cholecystitis, 28 % of the 7patients had no US findings diagnostic for gallbladder inflammation, mainly because of the absence of both a sonographic Murphy sign and gallbladder wall thickening [7]. Furthermore, diagnosis of acute cholecystitis must often be made with limited clinical information in patients with difficulties in communicating for reasons such as septic shock, dementia, brain damage, or use of sedative agents. The presence of a sonographic Murphy sign in these patients is difficult to evaluate correctly.

Increased pericholecystic attenuation on contrast-enhanced CT (CECT) is an objective and useful finding in the identification of acute cholecystitis [8, 9]. Some reports have supported the utility of contrast-enhanced US (CEUS) in differentiating between acute and chronic cholecystitis [10–12], by evaluating the intensity of contrast agent in the gallbladder wall. However, there have been no reports describing the diagnosis of acute cholecystitis using the contrast agent perflubutane (Sonazoid™; Daiichi Sankyo, Tokyo, Japan) in CEUS with time-intensity curve analysis along with an evaluation of the intensity of the contrast agent in the liver parenchyma adjacent to the gallbladder.

We hypothesized that increased enhancement of the liver parenchyma adjacent to the inflamed gallbladder is seen on CEUS, just as on CECT, and that time-intensity curve analysis can be useful to quantitatively express the findings. Thus, the purpose of this study was to evaluate whether CEUS with time-intensity curve analysis of the liver parenchyma adjacent to the inflamed gallbladder can improve the diagnosis of acute cholecystitis.

## Methods

### Patient selection

The Ethics Committee of our institution approved the study protocol (Kawasaki Medical School, registration number 1277). Informed consent was obtained from all patients before the injection of contrast agent.

From April to November 2013, 11 consecutive patients with acute cholecystitis (acute cholecystitis group) and 16 patients without acute cholecystitis (control group) were enrolled in this study. Patients with a focal spared area in the liver parenchyma adjacent to the gallbladder, as seen on gray-scale US imaging, and with portal vein embolism detected by color Doppler imaging, which may alter the focal perfusion, were excluded. The four males and seven females in the acute cholecystitis group had a median age of 68.0 years (range, 55–89 years). The diagnosis of acute cholecystitis was based on the TG13 criteria and involved surgery in seven patients and follow-up without surgery in four patients. All 16 patients in the control group underwent CEUS to search for metastatic liver tumors that could not be detected by gray-scale US. They were enrolled in this study under the criterion that no metastatic tumor was detected on segment 5 or 8 according to Couinaud’s classification. The 12 males and 4 females in the control group had a median age of 67.0 years (range, 40–86 years) and included patients with colon cancer (*n* = 8), gastric cancer (*n* = 5), lung cancer (*n* = 2), and duodenal cancer (*n* = 1).

### US technique and interpretation

**Fig. 1:**
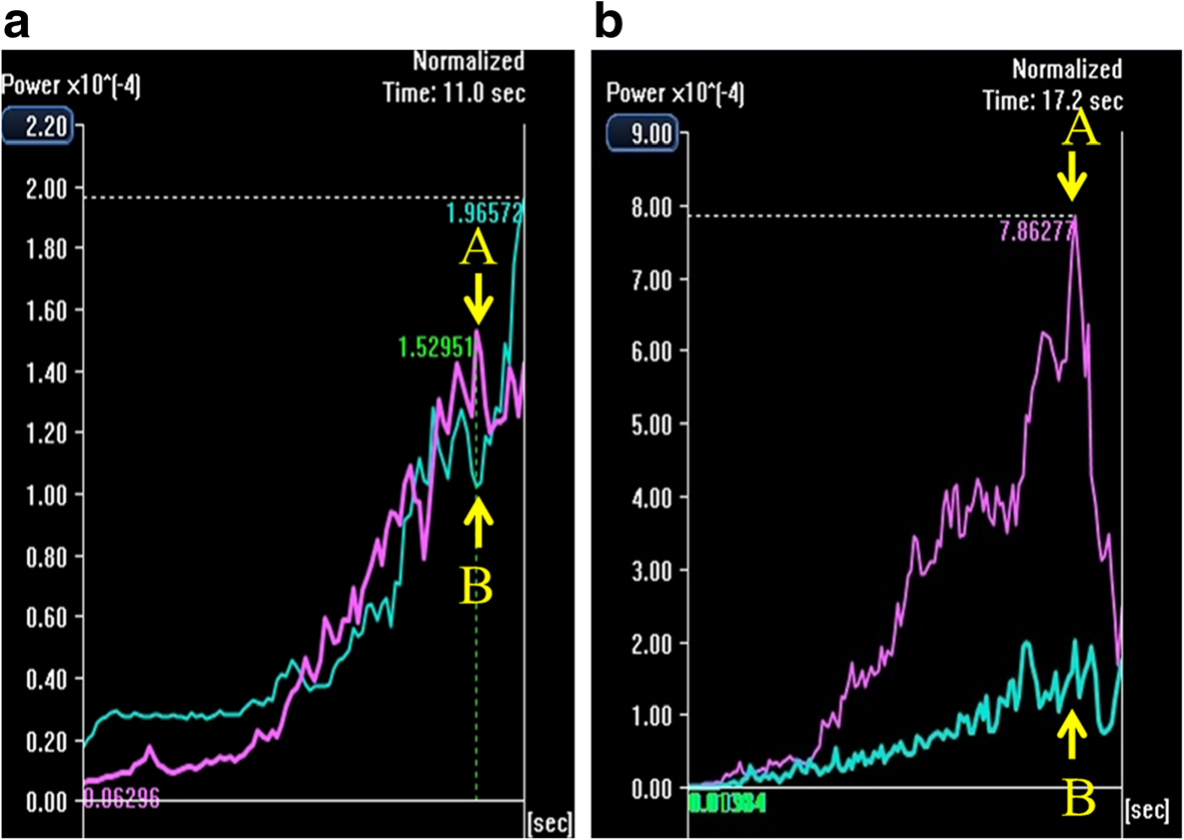
Time-intensity curves. **a,** Time-intensity curve of the non-cholecystitis group. The intensity ratio was defined as follows: the peak intensity of segment 5 (*arrow A*) divided by the simultaneous value of segment 8 (*arrow B*). Red line, segment 5; blue line, segment 8. **b,** Time-intensity curve of the acute cholecystitis group. The intensity ratio was defined as follows: the peak intensity of segment 5 (*arrow A*) divided by the simultaneous value of segment 8 (*arrow B*). Red line, segment 5; blue line, segment 8

**Fig. 2:**
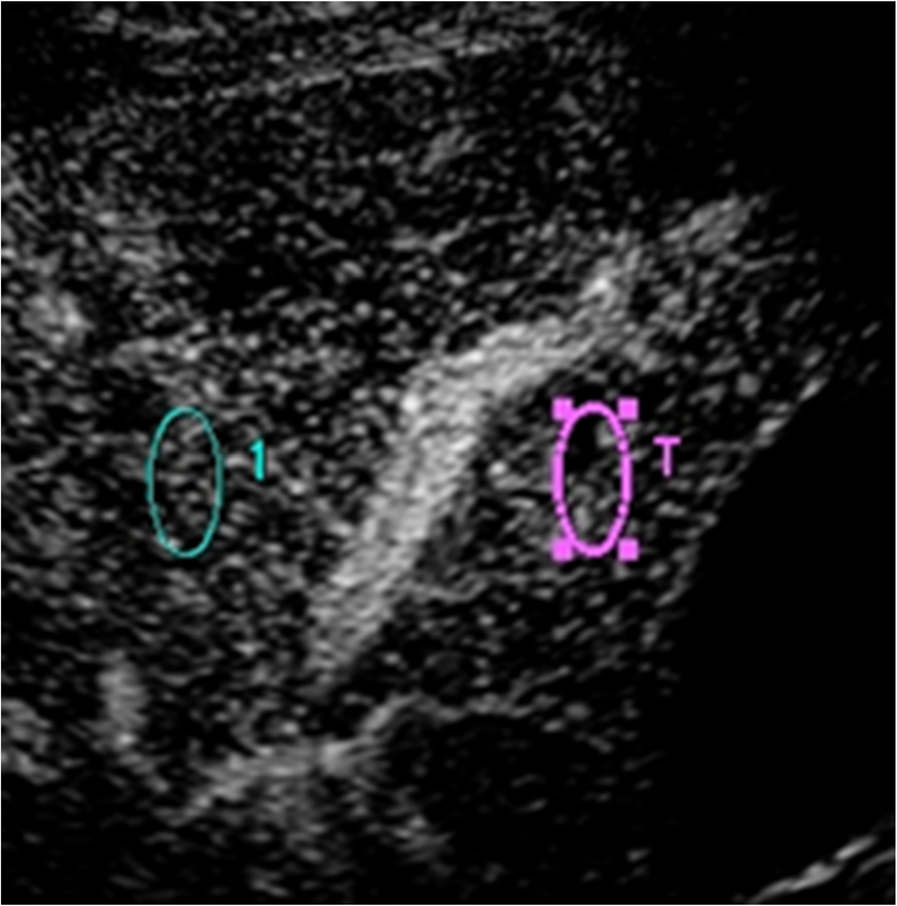
Region of interest (ROI) for two points. A pericholecystic point (*red circle*, segment 5) and another point at the same depth (*blue circle*, segment 8), avoiding the large vessels

All US examinations were performed with a diagnostic ultrasound system (TUS-A500; Toshiba, Tokyo, Japan) equipped with a 3.75-MHz transducer. No special patient preparations were undertaken. Gray-scale US was performed within 10 min in all patients. Sonazoid™ was injected intravenously (bolus, 0.015 mL/kg) followed by 10 mL of saline within 10 s. The gallbladder and liver parenchyma adjacent to the gallbladder were then scanned through the intercostal view using harmonic imaging with a low mechanical index (0.2–0.3) by one gastroenterologist (J.H.) with 21 years of experience in US. The raw imaging data, from the injection of the contrast medium to the beginning of enhancement of the main portal vein, were stored. Another physician (R.K.), with 3 years of experience in US, who was blinded to all clinical information then analyzed the data and constructed the time-intensity curve (Fig. 1a, b). To construct the time-intensity curve, the major axis of the region of interest (ROI) was set in segment 5 (the liver parenchyma adjacent to the gallbladder), and the control ROI was set in segment 8 on the same image (Fig. 2). The two ROIs were placed at the same depth. Neither ROI included relatively large vessels detectable by CEUS. The intensity ratio (IR) was defined as the peak intensity of segment 5 divided by the simultaneous value of segment 8. We considered that the IR would be suitable to demonstrate increased enhancement of the adjacent liver parenchyma by comparison with another point at the same depth that could be investigated simultaneously under the same conditions.

### Statistical analysis

The following patient characteristics were compared between the acute cholecystitis and control groups: sex, age, presence of liver cirrhosis, and clinical symptoms (fever or abdominal pain), white blood cell count, C-reactive protein level, liver enzyme concentrations (total bilirubin, aspartate aminotransferase, and alanine aminotransferase), gray-scale US findings (short-axis gallbladder diameter, presence of gallbladder stones, and presence of sonographic Murphy sign), and IR obtained by CEUS. All comparisons were performed using SPSS (version 19.0; IBM, Armonk, NY, USA). The Mann–Whitney *U* test for continuous values and Fisher’s exact test for categorical values were used to evaluate the significance of the differences between the two groups. A *p* value of <0.05 was considered to indicate a statistically significant difference. The correlation between the IR and the existence of acute gallbladder inflammation was analyzed using binomial logistic regression. A receiver operating characteristic (ROC) analysis was performed. The sensitivity and specificity of the IR for diagnosing acute cholecystitis were determined for each cut-off value using the resulting curve.

## Results

**Fig. 3:**
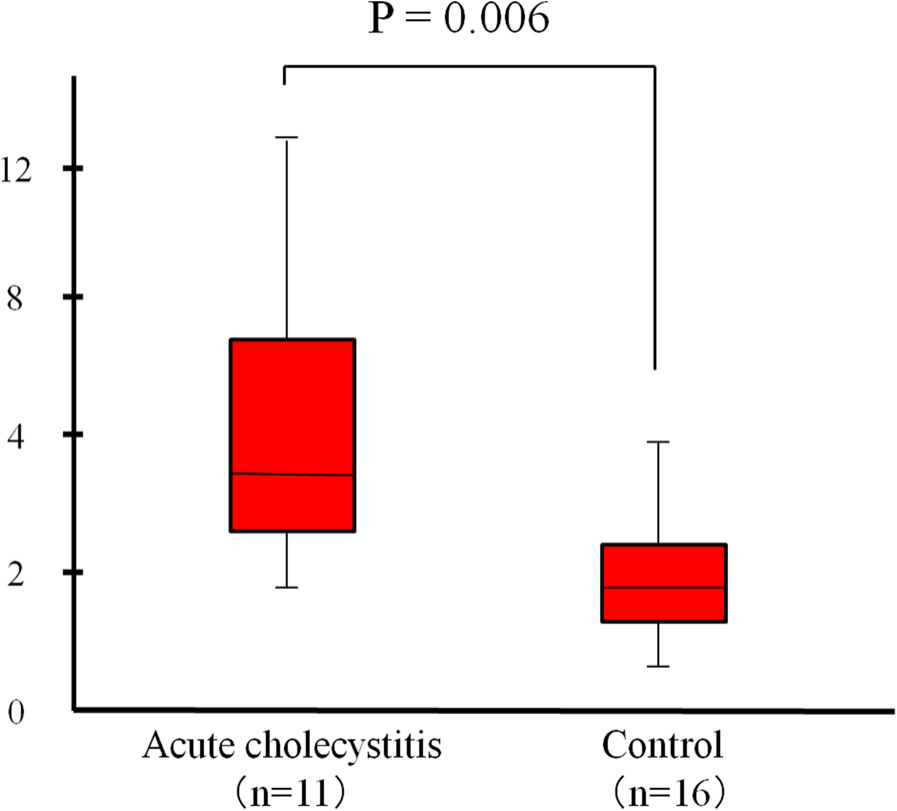
Intensity ratios (IR) of the two groups

The characteristics of all 27 patients in the two groups are detailed in Table 1. Most patients in the acute cholecystitis group had acute illness with abdominal pain (81.8 %, 9/11) and a positive sonographic Murphy sign (90.9 %, 10/11) consistent with acute cholecystitis. No patients in either group had liver cirrhosis. There were no significant differences in the laboratory test results, the short-axis gallbladder diameter, or the presence of gallbladder stones between the two groups. The IR obtained by CEUS was significantly higher in the acute cholecystitis group than in the control group (*p* = 0.006) (Fig. 3).

**Table 1:** Characteristics of the 27 patients in the acute cholecystitis and control groups

	Acute cholecystitis	Control	*p* value
Patients	11	16	
Male:female	4:7	12:4	0.061
Age, years	68.0 (64.0,81.0)	67.0 (56.5, 76.0)	1.000
Presence of liver cirrhosis, %	0 (0/11)	0 (0/16)	
Presence of fever (>37.5°), %	54.5 (6/11)	37.5 (6/16)	0.452
Presence of abdominal pain, %	81.8 (9/11)	31.3 (5/16)	0.018
White blood cell count, /μL	11010 (8615,13235)	6790 (5325, 8862.5)	0.054
C-reactive protein level, mg/dL	10.43 (3.77, 17.26)	0.56 (0.32, 4.89)	0.054
Total bilirubin level, mg/dL	0.90 (0.65, 1.20)	0.70 (0.50, 1.15)	0.452
AST level, IU/L	23.0 (17.0, 40.5)	40.5 (22.5, 63.3)	0.239
ALT level, IU/L	23.0 (14.5, 33.5)	34.0 (17.0, 53.5)	0.440
Short-axis gallbladder diameter, mm	30.0 (28.0, 35.5)	28.0 (22.0, 33.0)	0.198
Sonographic Murphy sign, positive, %	90.9 (10/11)	12.5 (2/16)	0.000
Gallbladder stones, positive, %	54.5 (6/11)	31.3 (5/16)	0.264
Intensity ratio	3.396 (2.90, 7.82)	1.595 (1.17, 2.50)	0.006

Data are presented as the median with the interquartile range (1st quartile, 3rd quartile)

*AST* aspartate aminotransferase; *ALT* alanine aminotransferase

Binomial logistic regression showed that the IR correlated significantly with the presence of acute gallbladder inflammation (*p* = 0.043). The odds ratio was 2.676 [95 % confidence interval (CI), 1.033–6.932].

**Fig. 4:**
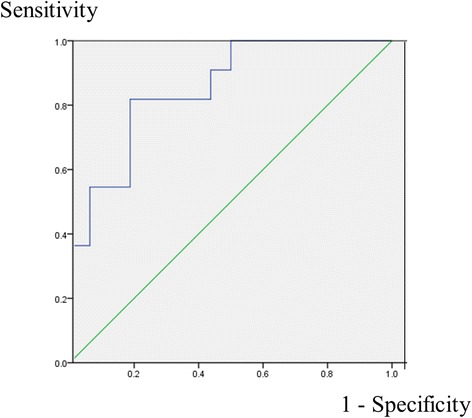
Receiver operating characteristic curve (ROC) of the intensity ratio for the diagnosis of acute cholecystitis. The area under the curve, indicating the diagnostic power, was estimated as 0.852 (95 % CI, 0.709–0.995)

Analysis of the ROC curve for the diagnosis of acute cholecystitis based on the IR (Fig. 4) showed that a cut-off value of 1.58 had a sensitivity of 100.0 % (11/11) with a 95 % CI of 67.9–100.0, and a specificity of 50.0 % (8/16) with a 95 % CI of 25.5–74.5. A cut-off of 2.72 had a sensitivity of 81.8 % (9/11) with a 95 % CI of 47.8–96.8, and specificity of 81.3 % (13/16) with a 95 % CI 53.7–95.0. A cut-off of 5.81 had a sensitivity of 36.4 % (4/11) with a 95 % CI of 12.4–68.4, and a specificity of 100.0 % (16/16) with a 95 % CI 75.9–100.0. The area under the curve was estimated as 0.852 (95 % CI, 0.709–0.995).

Five patients had an atypical IR: two in the acute cholecystitis group had a low IR (<2.72) and three in the control group had a high IR (>2.72).

## Discussion

US is the first-line morphologic examination technique for the diagnosis of acute cholecystitis [2, 3]. However, US findings consistent with acute cholecystitis are often seen in patients with conditions other than acute cholecystitis. Thickening of the gallbladder wall and free fluid around the gallbladder are not specific for gallbladder inflammation in patients with cardiac failure, renal failure, hepatic cirrhosis, hepatitis, hypoalbuminemia, or blockage of the lymphatic or venous drainage of the gallbladder [13]. Furthermore, the diagnosis of acute cholecystitis is difficult in patients who cannot explain their symptoms correctly (e.g., patients who are in a coma, are critically ill, have dementia, or have myeloparalysis). The development of a reliable and quantitative sonographic technique for the diagnosis of acute cholecystitis is therefore very important, both in these patients and in others.

Acute gallbladder inflammation causes increased blood flow from the cystic artery to the gallbladder wall. Transient and focally increased attenuation of the liver parenchyma adjacent to the inflamed gallbladder is a common CECT finding of acute cholecystitis, with a reported sensitivity of 82.4 % [8]. This finding can perhaps be explained by cholecystitis-induced hepatic arterial hyperemia and early venous drainage from the gallbladder [14, 15]. However, it is often difficult to safely transport critically ill patients to the radiology unit for CECT, especially those in the intensive care unit. Furthermore, in patients with concomitant renal dysfunction, CT contrast agents should be avoided because of their nephrotoxicity. Therefore, for the diagnosis of acute cholecystitis, we emphasize the utility of CEUS with the contrast agent Sonazoid™ as a bedside procedure for the detection of increased enhancement of the liver parenchyma adjacent to the inflamed gallbladder.

Adamietz et al. [10] used CEUS with SonoVue™ to examine 20 patients with acute cholecystitis and 8 with chronic cholecystitis. They reported that strong enhancement of the gallbladder wall was a very likely indicator of acute inflammation. However, our study is the first to demonstrate increased enhancement of the liver parenchyma adjacent to the inflamed gallbladder by CEUS together with time-intensity curve analysis. We used the IR, in which the peak intensity of the liver parenchyma adjacent to the gallbladder was divided by that of another point at the same depth, to avoid the interpatient variations caused by differences in the patients’ health. Our results showed that the IR was higher in patients with acute cholecystitis, in agreement with the Adamietz et al. [10]. The area under the curve, which represents the diagnostic power of this method, was estimated as 0.852. In our opinion, this represents a clinically acceptable diagnostic ability; thus, the IR obtained by CEUS with time-intensity curve analysis can facilitate the diagnosis of acute cholecystitis.

In the present study, 2 of the 11 patients in the acute cholecystitis group had an atypically low IR (<2.72). One of these patients, with an IR of 1.71, had histopathologically confirmed gangrenous cholecystitis. Accordingly, our method may have certain limitations in diagnosing gangrenous cholecystitis, in agreement with the results of a previous study [10]. The cause of the low IR (1.75) in the other patient remains unclear. In the control group, 3 of the 16 patients had a high IR (>2.72). The reason for this discrepancy is also unclear, but may have been due to the following: the mean IR among the other 13 patients in the control group was 1.52, indicating greater blood flow in segment 5 than in segment 8, even in patients without acute cholecystitis; this may be due to the normal venous return to segment 5 from the cystic artery. Additionally, anatomical variants of the cystic artery and the parabiliary venous system [16–19] may have contributed to these discrepancies. Furthermore, increased pericholecystic attenuation on contrast-enhanced CT is not pathognomonic for inflammation, as it is also observed in regions of focal fat deposition, cases of portal vein thrombosis, and similar conditions. Therefore, we excluded patients with a focal spared area in the liver parenchyma adjacent to the gallbladder by gray-scale US imaging and with portal vein embolism.

This study had several limitations. First, the number of patients was small. Second, we did not evaluate interobserver agreement. Finally, the patient selection of this study was based on the TG13. Therefore, the backgrounds of the two groups differed significantly in the clinical and gray-scale findings, which prevented an evaluation of the advantage of this method over the traditional diagnostic technique for acute cholecystitis using gray-scale imaging. This remains to be determined in further investigations.

## Conclusion

The IR obtained by CEUS with time-intensity curve analysis can generally demonstrate an increased enhancement of the liver parenchyma adjacent to the inflamed gallbladder.

## Abbreviations

*US*: Ultrasonography
*CEUS*: Contrast-enhanced ultrasonography
*CT*: Computed tomography
*CECT*: Contrast-enhanced computed tomography
*ROI*: Region of interest
*IR*: Intensity ratio
*ROC*: Receiver operating characteristic
*95% CI*: 95% confidence interval
